# Nanocarriers Used in Drug Delivery to Enhance Immune System in Cancer Therapy

**DOI:** 10.3390/pharmaceutics13081167

**Published:** 2021-07-28

**Authors:** Giovanna C. N. B. Lôbo, Karen L. R. Paiva, Ana Luísa G. Silva, Marina M. Simões, Marina A. Radicchi, Sônia N. Báo

**Affiliations:** Department of Cell Biology, Institute of Biological Sciences, University of Brasília, Brasília 70910-900, DF, Brazil; giovannalobo2012@gmail.com (G.C.N.B.L.); karendepaiva@gmail.com (K.L.R.P.); iza_gouvea@hotmail.com (A.L.G.S.); marinamesquita3007@gmail.com (M.M.S.); maradicchi.bep@gmail.com (M.A.R.)

**Keywords:** nanocarriers, immune system, nanobiotechnology, cancer

## Abstract

Cancer, a group of diseases responsible for the second largest cause of global death, is considered one of the main public health problems today. Despite the advances, there are still difficulties in the development of more efficient cancer therapies and fewer adverse effects for the patients. In this context, nanobiotechnology, a materials science on a nanometric scale specified for biology, has been developing and acquiring prominence for the synthesis of nanocarriers that provide a wide surface area in relation to volume, better drug delivery, and a maximization of therapeutic efficiency. Among these carriers, the ones that stand out are those focused on the activation of the immune system. The literature demonstrates the importance of this system for anticancer therapy, given that the best treatment for this disease also activates the immune system to recognize, track, and destroy all remaining tumor cells.

## 1. Introduction

Cancer is a major cause of global morbidity and mortality. It is a disease caused by a variety of factors, and its formation depends on several genetic and epigenetic aspects [1]. Malignant tumors have a specificity that affects other healthy cells in the body [2]. In order to develop more effective methods of diagnosis and treatment without harming the patient, various resources have been widely explored, and current treatment methods used for cancer control include chemotherapy, surgery, radiation, and biological therapies (immunotherapy and hormone therapy) [3,4,5].

However, these therapies have certain disadvantages and, being invasive, have side effects before and after treatment, making the patient uncomfortable. For example, the use of chemotherapeutic drugs can affect the normal and healthy growth of good cells and bring opportunities for tumor recurrence. In addition, resistance to various drugs may develop, and poor biodistribution results in a low concentration of these chemotherapeutic agents at the tumor site, which may reduce the therapeutic effect of anticancer drugs [6,7,8,9]. In this context, it is necessary to research and develop alternative beneficial and effective therapies for the drug delivery system.

Nanotechnology can increase the pharmacological properties of compounds commonly used in the treatment and diagnosis of cancer, which is why it has emerged as an innovative possibility for therapeutic intervention in cancer and in the distribution of drugs [10,11]. This can usually be achieved by different routes of administration, such as oral, nasal, transdermal, intravenous, etc. These nanocarriers can improve the effectiveness of the drug and reduce side effects. They can be encapsulated or used in combination with other drugs [12,13]. In addition, nano-scale transporters can protect drugs or any macromolecules (proteins, peptides, etc.) from degradation, reduce renal clearance, and provide sustained or controlled release kinetics, thereby increasing drug efficacy at steady-state therapeutic levels [14,15,16,17]. Their half-life in the blood improves the therapeutic index, solubility, and stability of the capsules, compared to conventional treatment methods (such as tablets, capsules, and injections) [18,19].

Nanocarriers are added as colloidal nanosystems loaded with therapeutic agents (anticancer agents or any macromolecules, such as proteins or genes), which allow drugs to selectively accumulate at the site of cancerous tumors [20,21,22]. As a result of their unique nanometer range, 1–1000 nm (drug administration is preferable in the 5–200 nm range), they are used for cancer treatment. The main and most promising nanocarriers in the literature are iron oxide, gold, polymers, liposomes, micelles, fullerenes (carbon nanotubes, graphene), dendrimers, quantum dots, and nanodiamonds [23,24,25]. Below, the following topics will be explored: lipidic, inorganic, and polymeric nanocarriers, targeting of nanomaterials by tumors, resistance of nanocarriers, and activation of the immune system.

## 2. Classification of Nanocarriers

**Figure 1:**
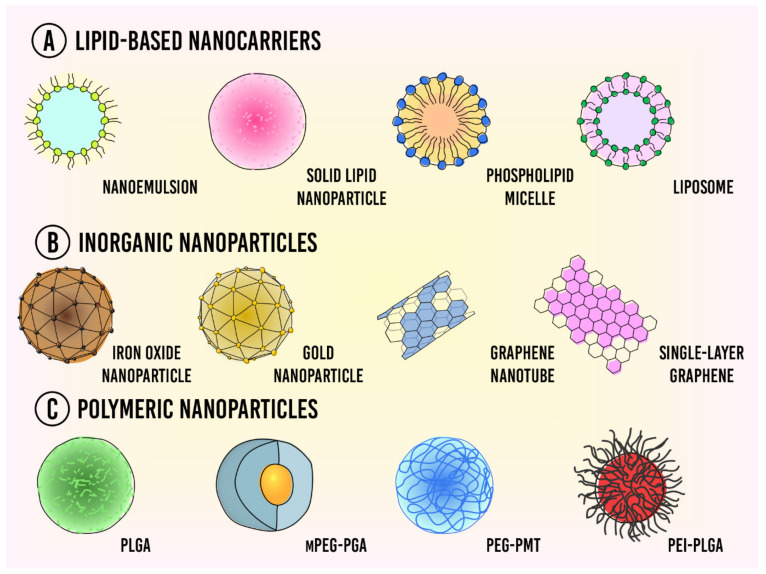
Types of nanocarriers used for drug delivery in cancer therapy. (**A**) Lipid-based nanocarriers; (**B**) Inorganic nanoparticles; (**C**) Polymeric nanoparticles.

Nanocarriers can be classified into three categories based upon the materials that they are made from (**A**) lipid-based nanoparticles, (**B**) inorganic nanoparticles, and (**C**) polymeric nanoparticles (Figure 1).

### 2.1. Lipid-Based Nanocarriers

Liposomes were the first nanotransporters advanced by Bangham [26] in 1965, and they include the first nanotransporter (DaunoXome ™) that was clinically approved by the FDA (Food and Drug Administration) for the transport of chemotherapeutic drugs in 1996 [27]. Lipid-based nanocarriers have emerged as a very promising, emerging, and rapidly developing tool for the delivery of various drugs with low solubility, bioavailability, and stability in recent decades [28,29]. Lipid nanocarriers allow the therapeutic load to be directed to the deep layers of the skin or even reach the blood circulation, making them a promising cutting-edge technology. Lipid nanocarriers refer to a large panel of drug delivery systems [30,31]. Lipid vesicles are the most conventional, and they are known to be capable of transporting lipophilic and hydrophilic active agents [32]. Others are designed with the objective of achieving a higher encapsulation rate and greater stability, such as solid lipid nanoparticles and nanostructured lipid nanocarriers [33]. The formulation of a liposomal drug improves the biodistribution and pharmacokinetics of a drug. This means that a higher concentration of the drug can be achieved within the tumors, while reducing the concentration of the drug in normal tissue [34,35]. Lipid-based nanocarriers include liposomes, nanoemulsions, solid lipid nanoparticles, and phospholipid micelles.

### 2.2. Polymeric Nanocarriers

Polymeric nanocarriers are synthesized from different types of natural and synthetic polymers that generally have good biocompatibility and biodegradability [36]. The advantages of these polymer nanomaterials compared to other nanocarriers include stability in various microenvironments, slow release of drugs due to polymer degradation, and their diversity in the types of polymers and types of drugs to be encapsulated [37,38]. The hydrophobicity and hydrophilicity within the polymer structure can be controlled to suit a variety of drug molecules [39]. Commonly used natural polymers include gelatin, dextran, albumin, chitosan, and alginate, and synthetic biodegradable polymers include polylactic acid (PLA), polyglycolic acid (PGA), copolymer of lactic acid and glycolic acid (PLGA), poly (ε-caprolactone) (PCL), polyalkylcyanoacrylate (PACA), poly (ethylene glycol) (PEG), poly (D,L-lactide-co-glycolide) (PLG), polyethyleneimine (PEI), poly (L-lysine), poly (Tian Particular acid), and others [40,41,42]. Gao (2021) [43] evidences the activity of reactive oxygen species (ROS)-responsive polymers for drug delivery systems, which may include polymers containing thioether, poly (thioketal), polymers containing selenium, tellurium, arylboronic acid/ester, aryl oxalate, and ferrocene; these are being widely investigated for anticancer therapy. The properties of polymerized NPs can be beneficial for the treatment of several potentially fatal diseases, including cancer, neurodegenerative diseases, cardiovascular diseases, and even viral infections and osteoporosis [44,45].

### 2.3. Inorganic Nanoparticles

Among the nanocarriers that are being developed for the diagnosis and treatment of cancer are inorganic nanoparticles, which may consist of iron oxide, silica, gold, and graphene, among other compounds [46]. There is greater difficulty in translating these types of nanomaterials (NMs) to clinical application, due to their lower biocompatibility and the lack of understanding of possible complications caused by their deposition in different organs, such as greater stability, less hydrophobicity, and non-microbial storage [47,48]. Despite these difficulties, the physical properties attributed to the constituent materials of inorganic nanoparticles make it possible to apply them in a variety of processes, for example, magnetic nanoparticles can be used for magnetic resonance imaging (MRI) or with magnetic targeting, while gold and silver NM can be used for imaging or heating during targeted treatment [49,50].

Similar to organic nanoparticles, inorganic NMs are also being studied as targeted drug delivery systems [45,46]. These important nanoparticles have gained attention in preclinical studies due to their potential for diagnosis and therapy in anticancer systems, with a variety of applications including tumor imaging, drug administration, and improvement in radiotherapy. Recent advances in nanotechnology demonstrate the importance of inorganic nanoparticles, given that they are internalized by cells through the endocytosis process [51] and can be composed of different materials, some of which are gold, oxide iron, and graphene [45,46,47,48,49,50].

Iron oxide nanoparticles (IONPs), composed of magnetite (Fe_3_O_4_) or maghemite (γ-Fe_3_O_2_), were initially developed and used as contrast agents for the detection of primary tumors and metastasis by magnetic resonance [51,52]. Due to the reduced nanometric size, usually varying between 10 and 20 nm, and their responses to magnetic fields, they have been developed to be associated with different drugs and to work as a drug delivery system [45,46,47,48,49,50,51,52,53]. Iron oxide nanosystems, once functionalized, have a wide field of biomedical application, being used preferentially for the treatment and diagnosis of cancer [54]. Studies demonstrate advantages in the use of IONPs for the greater uptake of intratumoral drugs, as shown in a study by Alphandéry et al. (2019) [53], which observed that the association of doxorubicin (DOX) with IONPs increased the accumulation of intratumoral drugs, when compared to free drug treatment against ovarian cancer models [55]. In another study, IONPs conjugated to vinblastine led to decreased viability of MCF-7 breast cancer cells when compared to treatment alone [56]. These processes can be improved when these NMs receive a targeting molecule, as in Nagesh et al. (2016) [57], which combined superparamagnetic iron oxide nanoparticles (SPIONs) with docetaxel, a chemotherapy, and an antibody against a specific membrane antigen present in prostate cancer (PSMA), and increased uptake and antitumor effectiveness compared to free drugs [57]. Associated with the drug delivery system, IONPs can also be used together.

Therapies can also be based on increasing local temperature, as in a study by Estelrich et al. (2016) [58], which used photothermal therapy with IONPs, resulting in the destruction of tumor cells (MCF-7 and MDA-MB-231) [58], or as contrast agents for imaging carcinomas, such as in Patel et al. (2016) [59]. The latter study used SPIONs associated with the anti-mesothelin antibody, which is a protein expressed in the membrane of different adenocarcinomas such as pancreas, lung, liver, sarcomas, to identify pancreatic carcinoma in xenographic models by MRI, which obtained very significant results when compared with the commonly used contrast agent (T2) [59].

Another group of inorganic nanoparticles that has been developed for the treatment of cancer are those composed of gold. Gold nanoparticles are being studied as an important mechanism for drug delivery, demonstrating better tumor targeting and, thus, reducing the adverse effects caused to patients by decreasing the doses of drugs used to treat various types of cancer [16]. In addition to the chemical properties of its surface, physical properties can also be exploited to act as contrast agents in imaging and as enhancers of anticancer therapy by increasing local temperature, leading to photothermal destruction of tumors [45,60]. A system proposed by Lee et al. (2017) [61], composed of doxorubicin linked to oligonucleotides and gold nanoparticles for therapy in cancer, has demonstrated promise in reducing colorectal cancer tumors [61]. In another study, the importance of the photothermal properties of gold nanoclusters covered by silica (AuNC ≅ SiO_2_) was demonstrated, showing the potential of these nanoparticles in the treatment of prostate cancer [62].

Graphene nanofibers, composed of a single layer of carbon atoms hybridized in sp^2^, have been used in biomedicine as imaging agents, anticancer therapy, and drug delivery systems [50]. The easy functionalization processes, excellent electrical conductivity, strong mechanical resistance, and high surface area make graphene an important agent to explore to compose the theragnostic NM used against cancer [50,63]. A study by Santos et al. (2018) [63] demonstrated the potential of the association of graphene oxide nanofibers with the methylene blue photosensitizing agent in the removal of xenographic breast tumors 4T1 through the combination of photodynamic and photothermal therapies [63]. As well as the potential for direct treatment against tumor cells, Deng et al. (2020) [64] evidenced the activation of antitumor macrophages in vitro and in vivo by means of graphene oxide nanofibers combined with polyethylene glycol (PEG), which is associated with near-infrared light irradiation (NIR) [64].

## 3. Resistance of Nanocarriers

Multi-drug resistance (MDR) in cancer can lead to the failure of chemotherapy and radiotherapy and has become one of the main obstacles to the success of cancer treatment [65]. Resistance to multiple drugs is the ability of tumor cells to exhibit simultaneous resistance to various structural and functional chemotherapeutic agents or radiation [66]. MDR is a complex process that completely modifies the behavior of tumor cells, generating changes in the targets of the drug, reduction of cell uptake, and the increase in drug efflux [67]. Some anti-MDR delivery platforms are based on drug delivery systems in multifunctional and responsive stimuli that can deliver drugs to cells and deliver at specific locations or times [68,69,70]. MDR can be treated with a combination of encapsulated cytotoxicity drugs and a chemosensitizer [71]. To overcome MDR, an ideal drug delivery system must release the drug quickly and completely into the cytoplasm, causing the intracellular concentration of the drug to be high enough to exceed the outflow of the drug and limit the concentration, thus inhibiting the cancer cells resistant to the drugs and killing them [72]. Specifically, the drug delivery system can control the drug release pattern and improve the accumulation of the drugs at targeting sites [71,73]. In this context, nanocarriers can achieve synergistic therapeutic effects and prove to have a stronger killing effect on cancer cells [74]. This can be seen in Alvandifar et al. (2018), in a study that constructed poly-lactid-co-glycolide (PLGA) carriers using SN38, an active metabolite of irinotecan, and verapamil, a MDR1 reversal agent, and they obtained a good result in cellular uptake and reduction of cell viability on MDA-MB-231 breast cancer cell line [71].

## 4. Targeting and Uptake of Nanomaterial to the Tumor

Nanobiotechnology, the creation of materials on a nanoscale applied to biology, has been developed and is gaining prominence due to early diagnosis and the targeted delivery of drugs to the tumor environment [75,76,77]. Controlled drug delivery systems have been extensively studied over the past few decades, resulting in more than 32,000 publications on colloidal systems [77]. Among these studies, the efficiency of the use of NMs in the detection and elimination of tumor cells in vitro and in vivo demonstrated that the clinical translation of these nanosystems is still statistically limited [78].

**Figure 2:**
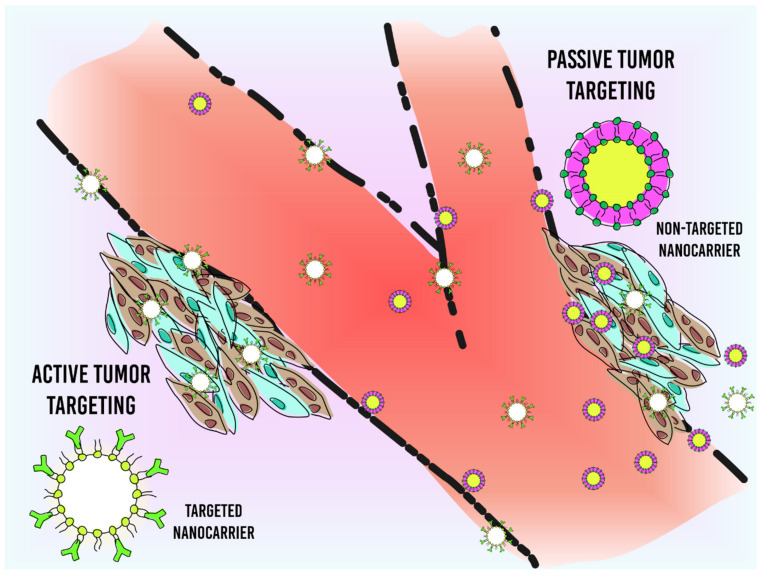
Schematic depiction of passive and active tumor targeting. Passive tumor targeting consists of the extravasation of nanomaterial by the increased vascular permeability of the tumor vessel associated with lower lymphatic drainage, which is commonly called the EPR effect. Active tumor targeting is the functionalization of nanomaterial with specific tumor ligands.

Currently, studies demonstrate that the physicochemical properties of NMs can influence the specific targeting for tumor cells, which can accumulate in this environment in a passive or active pathway [79,80] (Figure 2). The passive delivery of NMs is commonly called the enhanced permeability and retention (EPR) effect, which consists of the accumulation of these NMs in the tumor microenvironment through the problematic vasculature found in these locations. The increased permeability of the blood vessels associated with the tumor occurs due to the high metabolism of tumor cells and, consequently, of the endothelial cells associated with them, which during the angiogenesis process (formation of new vessels) do not complete the maturation process by forming vessels with irregular and larger fenestrations than normal sizes [46]. Unfortunately, NMs face a huge barrier until they find the tumor site and are easily eliminated from the bloodstream by the immune and reticuloendothelial system (RES) [81,82], which may be responsible for the unsatisfactory results found in the clinic. Indeed, Wihelm et al. (2016) [79] observed that from a database, only 0.7% of the administered dose accumulates at the tumor site, and Sindhawani et al. (2020) [83] demonstrated that the minimum dose necessary to achieve an efficiency of 93% uptake is one trillion nanoparticles [79,83].

Thus, research groups are looking for alternatives that result in an active and more targeted delivery of these NMs. For this, numerous functionalization processes are being developed aiming to evade opsonization, recognize the immune system and RES, and thus increase circulation time and the probabilities of reaching the tumor microenvironment [84,85,86]. In addition to the well-known functionalization with PEG and chemical agents, other mechanisms are also being used to increase the uptake of nanomaterials by tumors, such as modulation of the tumor microenvironment, targeting peptides and biological methods using cells, which will be described below [80].

The tumor microenvironment, a set of cells and molecules recruited and released by tumor cells, is an important component to be considered when targeting anticancer therapy [80]. The modulation of this environment, which can occur using chemical and physical reagents to improve tumor vasculature, assist in the reduction of tumor physiological changes, and alter components of the dense extracellular matrix associated with the tumor, has been shown to be an important contributing factor for therapies focused on treatment [86,87,88]. This was noted by Paris et al. (2020) [89], who used the combination of photodynamic and photothermal therapy with gold nanorods to increase nanoparticle (NP) uptake by the fibrosarcoma tumor and shut down the remaining tumor vasculature with an NIR laser [89].

In addition to the modulation of the tumor microenvironment, the use of ligands, such as tumor-binding peptides (IRGD) conjugated to the surface of NPs, has been shown to be promising in stimulating the transport of these nanomaterials into cancer cells and reducing drugs outside the target sites, consequently interfering in the patient’s quality of life [80]. Ni et al. (2015) [90] functionalized crystallized paclitaxel nanoparticles with IRGD (CAc) and demonstrated, in vivo, that only nanocrystals associated with IRGD were able to perform complete intratumor delivery and reach tumor stem cells [90].

Biological methods for improving the delivery of NPs are based mainly on the use of cell membranes or complete circulation cells that are tumoritropic, in order to increase and more effectively deliver NMs to the tumor environment [91,92]. This was observed in Wang et al. (2014) [93], in a study that used iron oxide nanoparticles with DOX linked to erythrocytes for the treatment of breast cancer in vivo, obtaining more satisfactory results when compared to the nanoparticle alone [91], and in the study of Piao et al. (2014) [94], which functionalized gold nanoparticles with an erythrocyte membrane, improving circulation time and, consequently, the treatment of 4T1 tumors [94].

Despite all the difficulties encountered in delivering NMs to the tumor environment, studies are being carried out to improve this process. Better results in the delivery of NMs to solid tumors may occur through the exploration of both the EPR effect and by active targeting strategies.

## 5. Strategies for Specific Delivery to Immune Cells

Macrophages compose the major population of infiltrating immune cells, and these cells are usually referred to as tumor-associated macrophages (TAMs) [94,95,96]. These TAMs usually overexpress macrophage mannose receptors (MMR), and nanocarriers can use this property to enhance the recognition and internalization of these macrophages [96]. On the other hand, these receptors are also found in healthy monocytes and macrophages and play a critical role in the immune system’s functions [97]. There is a receptor, CD206, that shows high expression on TAMs [98], and in humans, CD206 is more expressed in the M2 phenotype rather than the M1 phenotype [97]. Some studies have been successful after using the MMR receptor as a pattern of recognition, as seen with mannosylated poly (ethylene glycol)-conjugate nanocarriers, which can be a potential cell surface target on macrophages, including scavenger receptors such as the mannose receptor [99]. Anti-MMR nanobodies conjugated to polymeric nanogels were effective in delivering nanoparticles to MMR-expressing TAMs in vitro, ex vivo, and in vivo mice models. However, they are not the only functionalized nanocarriers that achieve specificity for TAMs; polymeric nanocarriers N-(2-hydroxypropyl) methacrylamide (HPMA) copolymers conjugated with Alexa Fluor 647 and folic acid (P-Alexa647-FA) are able to colocalize to CD11b + CD68 + TAMs in vitro and in vivo. In both primary and metastatic breast tumors, it has been shown that HPMA–copolymer folate conjugate could be localized in tumor-associated macrophages. The retinoid X receptor beta (RXRB) was also identified and validated as a macrophage-targeting peptide receptor binding on the surface of TAMs, with the anti-RXRB antibodies accumulated in TAMs [100]. Furthermore, red blood cell-derived nanovesicles (RDNVs) can be used as drug nanocarriers to specifically deplete macrophages; RDNVs are nanocarriers that can deliver drugs into macrophages both in vitro and in vivo [101].

Antigen-presenting cells (APCs) play an important role in activating the immune response. Among APCs, dendritic cells (DCs) are of paramount importance, as they are responsible for activating primary T lymphocytes and can effectively trigger the immune responses of cytotoxic T lymphocytes [101]. The first challenge in improving the activity of DCs is the specific binding of the nanocarriers to the target and, to do that, a nanoparticle formed by a cylindrical polymer with side chains of poly (2-oxazoline) was conjugated to an anti-DEC205. This was capable of specifically binding to and being uptaken by the DEC205 positive bone marrow derived dendritic cells (BMDCs) in the mouse model. When conjugated with an OVA-antigen, the same nanocarrier was able to stimulate the capacity of these BMDCs to activate and induce the proliferation of reactive CD8+ T-cells [102].

Other nanocarriers were designed to bind DCs, and some of these nanoparticles are based on HPMA [96], or formed by a protein cage named encapsulin (Encap) [102]. The Acetal-modified dextran-nanoparticles (Dex-NPs) were also able to bind to the dendritic cells [103,104]. Since the DCs are used as an antigen presenter, the development of vaccines using these nanocarriers involves different strategies to improve DC activity, such as the use of adjuvants or just the peptide of ovalbumin (OVA) protein, OT-1. They were all capable of activating CD8+ T, and one also activated CD4+ T and induced the proliferation of these cells, increasing the immune activity in the tumor area and suppressing tumor growth [102,103,104,105,106,107,108]. Other studies also showed that nanoparticles modified with mannose were able to increase the uptake of antigens by dendritic cells [109,110].

## 6. Tumor Microenvironment and Nanocarriers

The tumor microenvironment (TME) consists of cellular and non-cellular components that form the tumor’s niche. The non-cellular components are the extracellular matrix, cytokines, growth factors, small RNAs, DNA, chemokines, and extracellular vesicles. In addition to the cancer cells, there are also several other cell types, mesenchymal stromal, cancer-associated fibroblasts, adipocytes, pericytes, cancer stem cells, and the immune cells such as tumor-associated macrophages (TAM), dendritic cells (DC), T and B lymphocytes, neutrophils, and natural killer cells (NK) [111,112,113,114]. TME is characterized by oxidative stress, hypoxia, and acidosis and by interactions between these different cell types, which will cause the induction of angiogenesis, modulate the immune system, and lead to metastasis [115,116].

**Figure 3:**
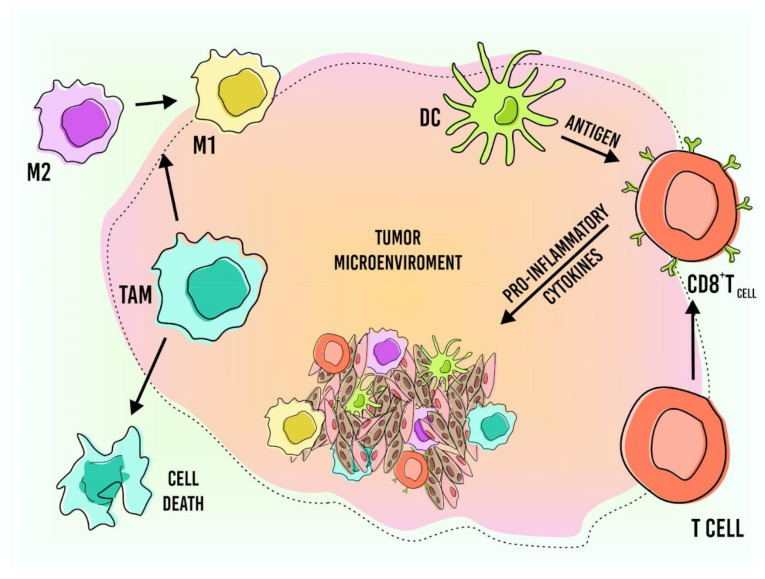
Immune cells associated with the tumor microenvironment and the possible outcomes after interaction with nanoparticles.

The outcome of altered metabolism in tumor cells in the tumor microenvironment directly affects the effectiveness of anticancer therapies (Figure 3). Thus, different research groups intend to develop new therapeutic strategies capable of modulating the TME. Among the new approaches is the use of nanocarriers in immunotherapy, which aims to change the immunosuppressive phenotype of TME [113,114,115,116,117,118,119,120]. Nanoparticles (NPs) can deliver drugs, antigens, and adjuvants that can manipulate immune cells at desired target sites (tumor and lymph nodes), in addition to evading pathophysiological barriers such as extracellular matrix, degradation of endonucleases, and renal clearance. Thus, the use of nanocarriers allows a more effective delivery of immunomodulators to the target tissue, which results in the amplification of the therapeutic potential of these molecules. The interactions of nanoparticles with the major immune cells, such as DCs, TAM, and T cells, are examined below. Studies on other cell types, such as B cells and natural killers, can be found in Thakur et al. (2020) [119,120,121,122,123,124,125]. Below, we introduce how nanotechnology addresses the cells of the immune system.

## 6.1. Tumor-Associated Macrophages (TAM)

Macrophages play an important role in controlling inflammatory responses, by releasing cytokines and presenting antigens [125]. Tumor-associated macrophages (TAM) are one of the essential modulators of the tumor environment [126]. As flexible cells, they can switch and be categorized in two major phenotypes: M1 represents macrophages that can exert phagocytosis in pathogens, prolong inflammation, provoke tissue damage, and have anti-tumoral activities. The other phenotype is M2, which is known to promote tumor development, and it is the type most found in the tumor microenvironment, because cancer cells induce macrophages to assume this phenotype by releasing several tumor-determining factors such as NF-κB, STAT3, and low pH values in the tumor microenvironment [127,128,129,130]. The M2 phenotype secretes a series of immunosuppressive cytokines responsible for the reorientation of infiltrating immune cells, and it also remodels the stroma [128]. It is responsible for tumor development and progression, survival, immune escape, wound healing, angiogenesis, metastasis, invasion, and resistance to chemotherapy [97,99,129], and it is also correlated with poor clinical outcomes [125]. So, therapies that can destroy M2 or restore it to the M1 phenotype are needed.

In this context, several researchers have studied the interactions that nanomaterials have with macrophages, since most circulating nanocarriers or tissue infiltrators are internalized mainly by phagocytic cells [131,132,133]. Thus, understanding how the properties of different nanoparticles can influence the polarization or inhibition of macrophages is extremely important for the use of this technique, in order to direct these cells to nanotherapeutic formulation and alter the TME in a more permissive environment for the function of the immune cells [134]. In this approach, organic, inorganic, and hybrid nanoparticles have been extensively explored to verify their potential to modulate the TME [134,135,136]. As an example, a drug nanocarrier based on the copolymer of lactic acid and glycolic acid (PLGA) loaded with methotrexate (MTX), covered with polyethyleneimine (PEI) and hyaluronic acid (HA), and combined with a PD-L1 antibody, was developed to investigate the immunomodulatory effects on breast cancer TME. The nanoparticles of PeiPLGA-MTX were able to downregulate the STAT3 and NF-κB genes, which resulted in the change of the M2 (CD163) to M1 (CD68) phenotype and reduced the levels of IL-10, TGF-β, and CCL22. Thus, NPs from PeiPLGA-MTX were effective in polarizing macrophages and modified the course of disease development [128]. Another nanoparticle, Man-PEG-Lipo, showed a different approach: the great difference is that it induced not only the polarization of the M2 to the M1 phenotype but also promoted the polarization of the M0 to the M1 phenotype. The in vivo model presented tumor growth inhibition, which may be attributed to the impact of Man-PEG-Lipo on the polarization of TAMs [137].

Another example is inorganic particles, such as gold nanoparticles (AuNPs) developed by Kim et al. (2020) [138], in a study that evaluated the effect of this nanomaterial with radiotherapy (RT) on M2 TAMs of tumors. AuNPs conjugated with the CD163 antibody, and after being coated with silica (CD163-GNPs), they were tested in in vitro and in vivo models. The analyses revealed that a greater number of CD163-GNPs were absorbed by M2 macrophages than those of type M0 or M1 and were able to alter the phenotype of TAMs. In addition, the polarization of type M2 macrophages in tumors after combination treatment with CD163-GNPs increased the efficiency of RT [138]. In addition, superparamagnetic iron oxide nanoparticles (SPIONs) can act as co-stimulators during macrophage activation and alter their polarization by shifting their M2 phenotype without significantly reducing cell viability of macrophages [131]. 

Therefore, NPs capable of directly reaching the immune cell, such as TAMs in the tumor microenvironment, can effectively increase local and systemic antitumor immunity [133,134,135,136,137,138,139,140,141,142]. Nanocarriers can also be used as a platform to deliver genetic information used to reprogram the TAMs. This is seen with the in vitro-transcribed (IVT) mRNA used in TAMs; after serial administration of IRF5/IKKβ-encoding nanoparticles, two potent pro-inflammatory agents, the nanocarrier can substantially reduce tumor progression and also reduce the density of M2-like macrophages in a mouse tumor model, along with increased numbers of inflammatory myeloid cells with distinct M1-type transcriptional profiles [143]. A microRNA, the miRNA-155 (miR155), is characterized as a pro-inflammatory miRNA, and it can enhance the M1-like macrophage activation by decreasing inhibitors of pro-inflammatory responses, so a nanocarrier to deliver this miRNA was developed, which was constituted by a lipid-coated calcium phosphonate conjugated with mannose (CaP/miR ≅ pMNPs). These nanoparticles were able to reactivate TAMs and reprogram their functions, thus reversing the immunosuppressive tumor microenvironment, inhibiting tumor growth, and reducing tumor sizes and weight after systemic administration in a tumor-bearing mouse model [130].

In order to inhibit TAM, different nanocarriers have been used. A virus-based delivery vehicle, the icosahedron cowpea mosaic virus (CPMV), conjugated with a zinc ethynylphenyl porphyrin (Zn-EpPor), a photosensitizer, PS-CPMV demonstrated a significant improvement in the effective elimination of both macrophage and tumor cells, with differences in uptake between the M1 and M2 populations observed [143]. Platinum (Pt)-prodrug conjugated small particles and BLZ-945, a small molecule inhibitor of colony-stimulating factor 1 receptor (CSF-1R) of TAMs, were capable of codelivering nanocarriers. As well as inducing the apoptosis of tumor cells, it also modulated the tumor immune environment and eventually increased the antitumor effect of CD8+ cytotoxic T cells through TAM depletion and exhibited properties to suppress tumor growth, inhibit metastasis, and prolong the survival of the mice model [126]. The p-(aminomethyl) benzoic acid (PAMB)/doxorubicin (DOX) was capable of specifically decreasing TAM. PAMB/DOX effectively suppressed the primary tumor growth and inhibited tumor migration, invasion, and metastasis formation. It also improved the tumor microenvironment by inhibiting ECM degradation, neovascularization, and tumor cells’ escape [129]. 

## 6.2. Dendritic Cells (DC)

In an attempt to use the immune cells to attack the tumor, an important agent is antigen-presenting cells (APCs), such as B-lymphocytes, macrophages [144] and, the most potent, dendritic cells (DCs) [145]. DCs are able to capture, process, and present tumor-associated antigens (TAA) [146], which induce and activate a specific tumor immune response by presenting these antigens to T cells [105,110], as T helper cells (CD4+ T cells) and cytotoxic T cells (CD8+ T cells) [105] that can secrete cytokines to induce tumor rejection, such as IFN-γ [146], which can trigger and control an antitumor immune response [146]. Due to the immunosuppression generated by changes in the tumor microenvironment, there is no effective antigen presentation for T cells. Thus, immunotherapy has made use of DC-based vaccines, and one of them has already been approved by the Food and Drug Administration (FDA) of the United States for the treatment of prostate cancer [107]. DC vaccines consist of the ex vivo pulse of dendritic cells with antigens associated with the tumor. Right after the capture of antigens, the DCs reinserted in the patients must migrate to the lymph nodes and, through the presentation of antigens to the T cells, reactivate the antitumor immune response. However, results in clinical trials were unsatisfactory, and less than 10% of DC-mediated immunotherapy was reported to be effective. The low rate of migration of dendritic cells applied to the lymph nodes, together with the lack of efficient antigen loading and low maturation of DCs, are the main reasons why vaccines are failing to activate T cells and induce the immune system to fight cancer [147,148]. In this context, several nanocarriers are being designed to modulate dendritic cells, to reduce the limitations present in this technique [118]. Several nanoparticle systems can facilitate the delivery of the antigen in the cytosol and achieve cross-presentation. NPs are also able to protect the antigen from degradation by enzymes that circulate in the blood, which significantly increases the efficiency of intracellular uptake of the antigen through endocytosis [148].

Current studies have shown that organic nanocarrier platforms have been successful in delivering antigens and stimulating immune responses. Recently, it was demonstrated that polyethylenimine nanoparticles (PEI) with the ovalbumin (OVA) antigen model, functionalized with mannose, increased the targeting for DCs. In addition, there was an accelerated endosomal leak and improved presentation of the MHC-I antigen for cancer immunotherapy [149]. Although most vaccines use OVA as an antigen, cancer-specific antigens can be used. To deliver DNA vaccines specifically to the antigen-presenting cell, the use of gold nanoparticles (AuNPs) decorated with a thiolate ligand (SGSH) and a melanoma antigen (MARTI1) encoded DNA vaccine (Au-SGSHpCMV-MART1) was efficient in vitro and in vivo. This presented immunization against melanoma in a murine model for 180 days limited tumor growth, increased survival in mice, and presented a DC-mediated improvement in the CD8+ T cells by elevating the IFN-γ secretion, showing that inorganic nanocarriers can be associated with antigens and activate the immune system [144].

Despite the high potential of DCs, some immunoregulatory factors, for instance IDO (dioxygenase), can make the antitumor immune response become insufficient. Thus, to outdo the negative activity of the immunoregulator, a nanoparticle using gold nanorods (GNR) as a carrier of siRNA for IDO (siIDO) and also using mannose to target the DCs (man), man-GNR-siIDO, was used as a knockdown for the IDO gene, and it resulted in an effective silencing particle. This was effective both in vitro and CD11b +, promoted the DC maturation and upregulated T cell proliferation, also decreasing the apoptosis of CD4+ T and CD8+T cells with increased tumor-specific cytotoxicity [105]. It is not only siRNAs that are delivered to DCs; plasmid DNA carried by mannosylated liposomes can also be useful to encode specific modulators and is able to generate tumor-specific cytotoxic T lymphocytes by DC mediation, providing a high anti-metastatic potential [110]. Thus, studies corroborate the idea that the construction of nanovaccines against cancer has the potential to promote the maturation of DCs, since after phagocytosis, they released antigens in a controlled manner, leading to an increased cellular and humoral immune response [111,150,151,152].

## 6.3. T Cells

The tumor microenvironment is characterized by being an immunosuppressive environment, which prevents an immune system response against cancer. Immunotherapy seeks to reverse this scenario through the activation of different pathways, one of which is the reactivation of T cell-mediated immunity. Thus, the induction of a strong cytotoxic T cell response is an important prerequisite for successful treatment against tumors [153,154]. Under these circumstances, the adoptive transfer of T cells derived from patients with chimeric antigen receptors (CARs) is a potential therapeutic strategy in the treatment of cancer. CAR T cells can initiate the production of antitumor cytokines and proliferate exponentially, which results in an amplified immune response and the elimination of tumor cells. Although this technique has promising results, there are still limitations, such as the lack of efficient targeting to the tumor, limited response rate, laborious manufacturing processes, and high cost. Thus, nanocarriers have emerged as an alternative means to overcome these obstacles and amplify the use of this technique [117,155].

Studies show that nanocarriers can efficiently introduce CAR genes into T cell nuclei, inducing tumor recognition and regression [117,156,157,158,159]. The experiments carried out by Bai et al. (2020) [160] demonstrated that polymers of multivalent aptamers could fulfill the CAR-T function, since they were able to increase the proliferation of T cells, reverse the inhibitory effect of the secretion of antitumor cytokines, and inhibit the growth of mouse melanoma B16 cells both in vitro and in vivo [157]. The use of nanotechnology for the transient induction of CAR in T cells can also mitigate the side effects caused by the current method of using viral delivery vectors for the reprogramming of T lymphocytes [145]. The use of ionizable lipid nanoparticles (LNPs) for the delivery of CAR mRNA to primary human T cells was described by Billingsley et al. (2020) [159], which pointed out the use of this platform as promising for the expansion of T cell engineering. The results of the study showed the ability of LNPs to deliver mRNA from the CAR to primary T cells and generate functional CAR T cells that have the potential to induce cancer cell death [159]. Furthermore, another approach to reprogramming T cells is to use nanocarriers to deliver drugs, antibodies, and interleukins, as they can protect T cells from immunosuppression and activate CD8+ T cells [160,161,162,163,164].

NPs can deliver drugs and monoclonal antibodies that block negative feedback, such as cytotoxic protein associated with T 4 lymphocyte (CTLA-4), programmed cell death protein 1 (PD-1), and indoleamine 2,3-dioxygenase (IDO) [165,166,167]. The targeted and effective delivery of these compounds to CD8+ T cells is achieved by modifying the surface of the nanoparticles with the target ligand or antibody. An example of an NP that has been widely used to transport immunomodulating agents is the copolymer of lactic acid and glycolic acid (PLGA), due to low immunogenicity and good biodegradability [163,167,168,169]. Overall, the studies presented demonstrate that nanocarriers are a promising platform for cancer immunotherapy. Examples of nanocarriers used to modulate the tumor microenvironment are summarized in Table 1.

## 7. Conclusions and Future Prospects

Years of research have investigated therapeutic alternatives for treating cancer, including surgery, radiation therapy, chemotherapy, hormonal therapy, and target therapy, which are provided according to their type, stage, and location. Currently, it is known that the common therapeutic approaches applied to the treatment of this disease, despite promoting a good prognosis for the patient, cause damage to healthy tissues, incomplete eradication of tumor cells, and adverse effects on patients. Observing these various problems associated with current treatments, new therapies have been sought that are non-invasive and have low systemic toxicity, such as nanobiotechnology. The nanotechnology field has established recently promoted cancer treatment methods. Several nanocarriers studied in this field have allowed researchers to overcome the limitations of conventional therapies, thus increasing their efficiency in guiding the delivery of these therapeutic agents.

The studies reinforced the concept that nanocarriers can be developed to promote the recruitment of immune cells and overcome the anergy of tumor-specific T cells by blocking immunosuppressive pathways. The use of nanocarriers is a promising strategy, because it is possible to have a modular response in the TME, activate an accurate CTL response, and improve antitumor efficacy. In this context, immunotherapy combined with nanoparticles will have a significant impact on clinical performance to enhance the immune system in cancer therapy. However, there is still a need for studies to gain a deeper understanding of the mechanisms underlying the immune system and the safety profiles of nanoparticles, to ensure effective delivery of nanocarriers to the target tissue and avoid toxicity.

## Figures and Tables

**Table 1:** Nanocarriers used to modulate the tumor microenvironment.

NP Platform	Immunotherapeutic Agent	Function	Ref.
**Organic NP**			
iDR-NCs	shRNA	CD activation	117
Peptide assembling	DPPA-1; NLG919; IDO inhibitor	To block immune checkpoints and tryptophan metabolism	122;164
Protein nanogels (NGs)	IL-15; Anti-CD45	Active T cell	155
Lipid-CaO 2	CaO 2	TME modulation (hypoxia)	114
Man-PEG-Lipo	Curcumin	Macrophage polarization	136
CaP/miR@pMNPs	Genetic information (miRNA 155)	Macrophage polarization	129
Polyethylenimine (PEI)	OVA	CD activation	148
Aptamer nanoparticles	Genetic information	Activate T cells	156
Lipid nanocapsules (NCs)	mRNA	Activate T cells	158
**Polymeric NP**			
PeiPLGA-MTX	Methotrexate	Macrophage polarization	127
PGLA	IL-12	Activate T cells	164
PEG-PMT	Doxorubicin	TME modulation	43
mPEG-PGA	Doxorubicin	TME modulation	41
**Inorganic NP**			
Iron oxide NPs	Intrinsic therapeutic effect	Macrophage polarization	130;101
CD163-GNPs	Intrinsic therapeutic effect	macrophage polarization	137
Gold nanorods	siRNA specific for IDO	CD activation	146
Au-SGSH	genetic information	CD activation	143
Platinum	BLZ-945	Macrophage polarization	124

NP = Nanoparticles
